# Supplementary material for: The impact of stopping and starting indoor residual spraying on malaria burden in Uganda
Source: Nat Commun. 2021 May 11;12:2635. doi: 10.1038/s41467-021-22896-5 (PMC8113470; doi:10.1038/s41467-021-22896-5)
Supplement: Supplementary file 1 — Supplementary Information [file 41467_2021_22896_MOESM1_ESM.pdf]

Supplementary Fig 1. Timeline indicating the dates of vector control campaigns, baseline and evaluation periods for the 14 study sites.

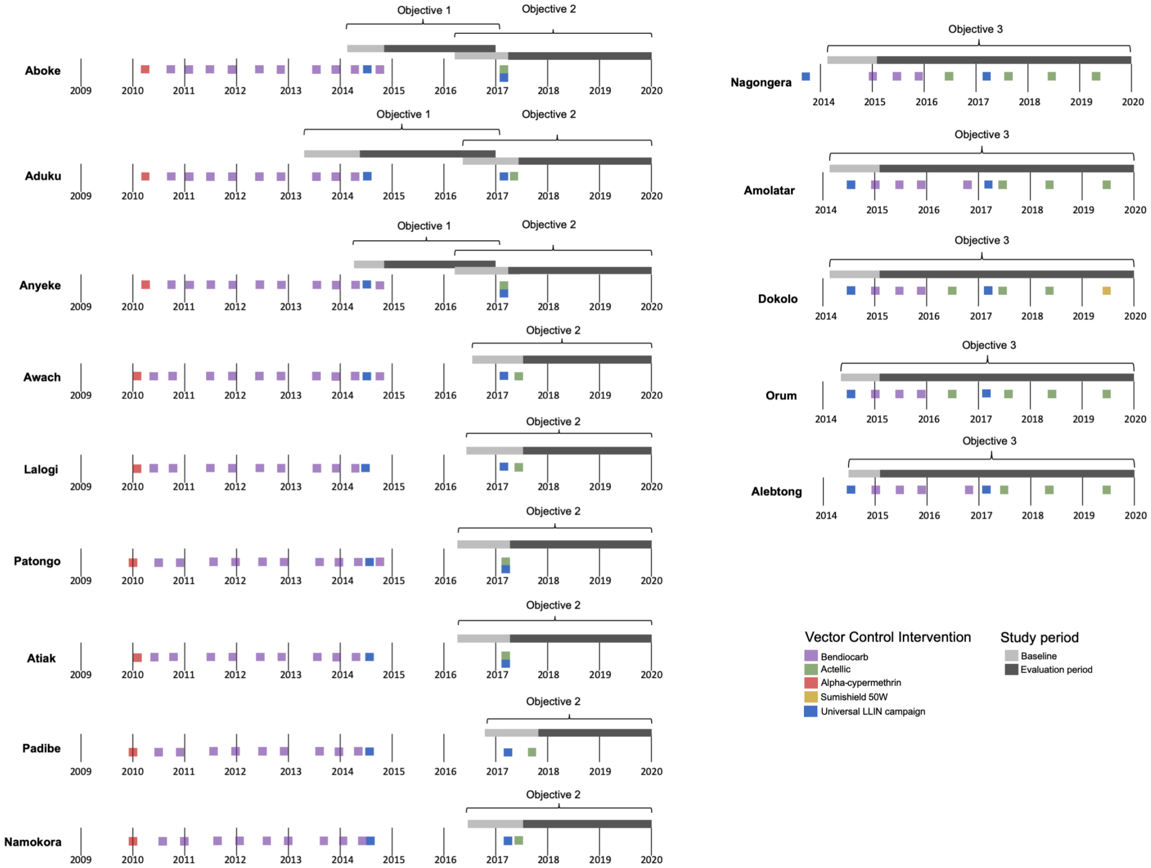

Supplementary Fig 2. Case counts over time for the three study sites included in the analysis for study objective 1.

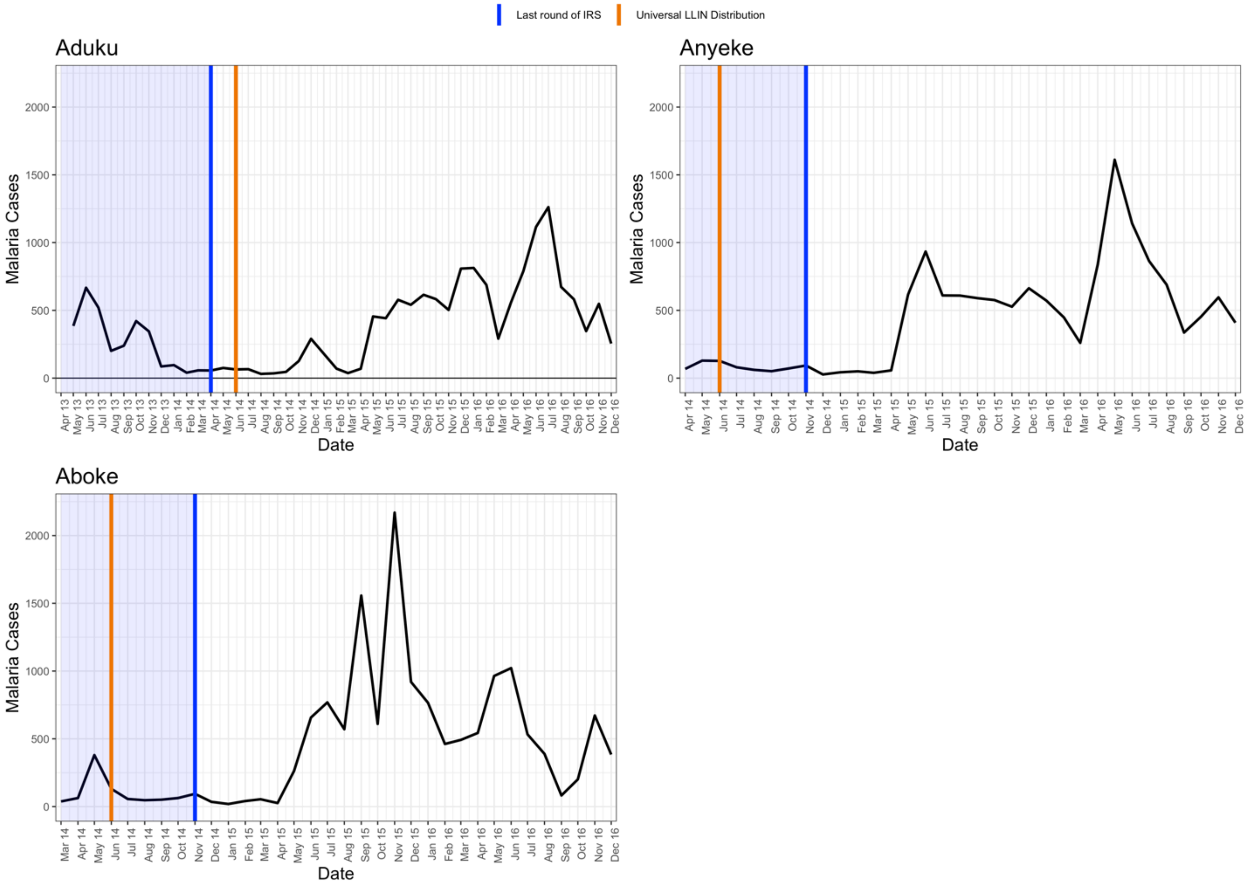

Supplementary Table 1. Coefficients and 95% confidence intervals for study objective 1. P-values are 2-sided and correspond to t-test from regression models. No adjustment for multiple comparisons was made.

Variable	**OR**	**pvalue**	**LL**	**UL**
Months since IRS stopped				
0	REF	0.001	0.094	0.527
1	0.223	0.002	0.117	0.604
2	0.266	0.242	0.240	1.434
3	0.587	0.244	0.272	1.392
4	0.615	0.048	0.218	0.992
5	0.465	0.445	0.642	2.746
6	1.327	0.098	0.880	4.586
7	2.009	0.040	1.040	5.091
8	2.301	0.000	2.483	11.925
9	5.442	0.000	3.790	20.128
10	8.734	0.000	2.166	10.122
11	4.683	0.000	2.925	13.170
12	6.207	0.001	1.921	10.702
13	4.535	0.000	1.952	10.785
14	4.588	0.000	3.116	17.264
15	7.334	0.000	3.687	17.487
16	8.030	0.000	3.624	16.648
17	7.767	0.000	3.129	13.562
18	6.514	0.000	2.411	11.714
19	5.314	0.000	2.158	10.762
20	4.819	0.000	3.833	18.727
21	8.472	0.000	1.946	8.524
22	4.073	0.000	2.327	10.088
23	4.845	0.000	2.234	9.625
24	4.637	0.001	1.710	8.683
25	3.853	0.001	2.358	23.600
26	7.459	0.000	2.487	23.305
27	7.613	0.001	2.080	19.145
28	6.310	0.024	1.191	11.658
29	3.726	0.025	1.184	12.104
30	3.785	0.122	0.775	8.799
31	2.611	0.516	0.956	1.023
Precipitation (1 month lag)	0.989	0.516	0.956	1.023
Proportion of tests RDT	0.915	0.645	0.627	1.336
Count of patients not suspected of having malaria	1.000	0.026	1.000	1.001
Calendar month				
January	REF			
February	0.437	0.103	0.161	1.184
March	0.363	0.032	0.144	0.917
April	0.528	0.169	0.213	1.312
May	1.640	0.237	0.722	3.724
June	1.846	0.171	0.767	4.443
July	1.277	0.431	0.695	2.344
August	0.604	0.280	0.242	1.509
September	0.638	0.324	0.261	1.558
October	1.035	0.935	0.450	2.378
November	1.223	0.649	0.514	2.913
December	1.126	0.809	0.430	2.947
Constant	126.954	0.000	54.478	295.851

Supplementary Fig 3. Adjust IRR (the measure of center for the error bars) and 95% confidence intervals (the error bars) for study objective 1 with raw laboratory-confirmed case counts as model outcome.

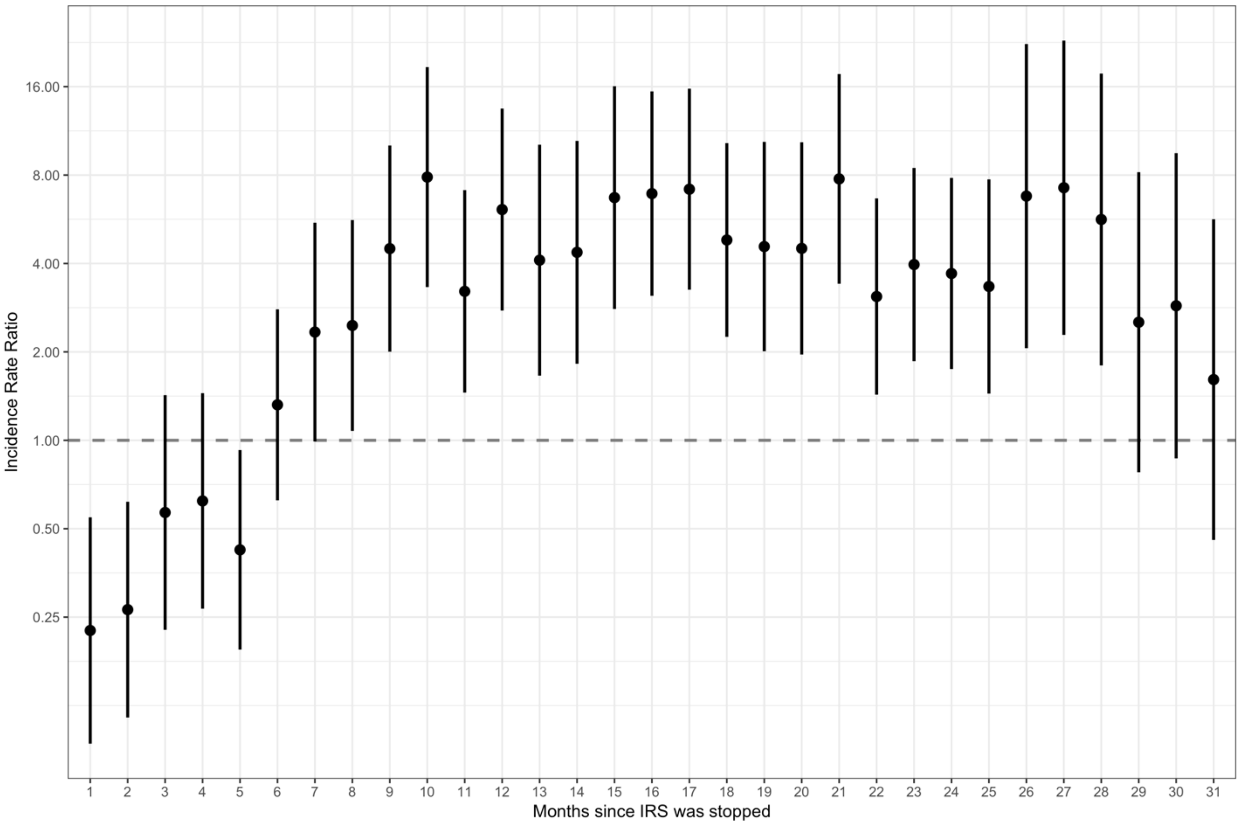

Supplementary Fig 4. Case counts over time for the nine study sites included in the analysis for study objective 2.

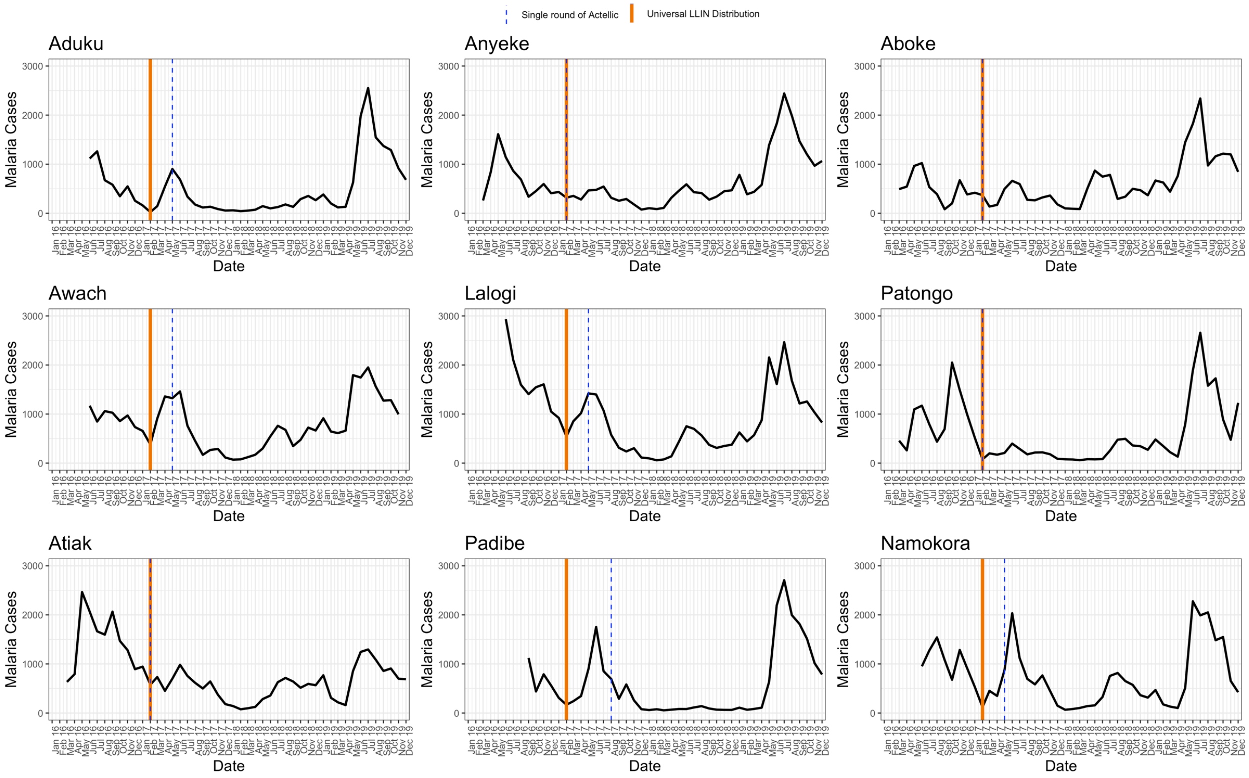

Supplementary Table 2. Coefficients and 95% confidence intervals for study objective 2. P-values are 2-sided and correspond to t-test from regression models. No adjustment for multiple comparisons was made.

Variable	**OR**	**pvalue**	**LL**	**UL**
Months since single round of IRS				
0	REF			
1	0.706	0.033	0.513	0.973
2	0.529	0.000	0.385	0.727
3	0.373	0.000	0.272	0.511
4	0.262	0.000	0.190	0.361
5	0.293	0.000	0.212	0.405
6	0.274	0.000	0.198	0.378
7	0.208	0.000	0.151	0.286
8	0.203	0.000	0.147	0.281
9	0.194	0.000	0.140	0.268
10	0.163	0.000	0.118	0.225
11	0.180	0.000	0.130	0.249
12	0.205	0.000	0.148	0.283
13	0.188	0.000	0.136	0.260
14	0.339	0.000	0.245	0.469
15	0.351	0.000	0.254	0.486
16	0.265	0.000	0.191	0.369
17	0.315	0.000	0.227	0.438
18	0.412	0.000	0.298	0.570
19	0.455	0.000	0.330	0.627
20	0.595	0.002	0.429	0.826
21	0.703	0.033	0.509	0.971
22	0.750	0.072	0.549	1.026
23	1.090	0.593	0.795	1.493
24	1.162	0.351	0.848	1.591
25	0.976	0.880	0.711	1.340
26	1.154	0.376	0.840	1.587
27	1.126	0.464	0.819	1.548
28	1.037	0.822	0.756	1.423
29	1.181	0.341	0.839	1.662
30	0.953	0.783	0.678	1.340
31	0.963	0.838	0.673	1.378
32	0.865	0.540	0.545	1.374
33	0.817	0.395	0.513	1.302
34	1.445	0.120	0.908	2.298
Precipitation (1 month lag)	0.988	0.044	0.977	1.000
Proportion of tests RDT	2.351	0.000	1.742	3.174
Count of patients not suspected of having malaria	1.000	0.860	1.000	1.000
Calendar month				
January	REF			
February	0.633	0.003	0.470	0.851
March	0.742	0.044	0.554	0.992
April	0.967	0.784	0.759	1.232
May	2.103	0.000	1.587	2.786
June	3.035	0.000	2.271	4.055
July	2.636	0.000	2.049	3.391
August	1.984	0.000	1.462	2.691
September	2.026	0.000	1.515	2.709
October	2.224	0.000	1.689	2.929
November	1.848	0.000	1.388	2.460
December	1.198	0.216	0.900	1.594
Constant	300.471	0.000	195.082	462.794

Supplementary Fig 5. Adjust IRR (the measure of center for the error bars) and 95% confidence intervals (the error bars) for study objective 2 with raw laboratory-confirmed case counts as model outcome.

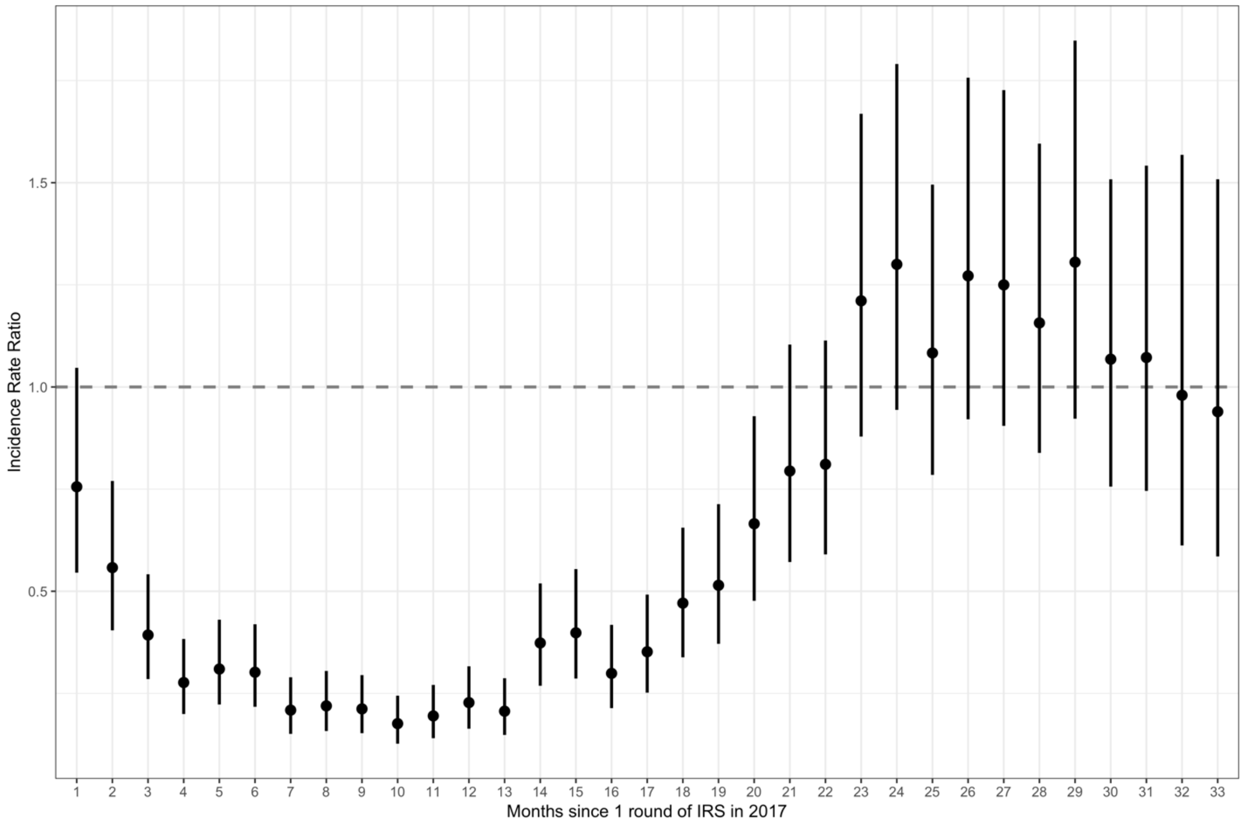

Supplementary Fig 6. Case counts over time for the five study sites included in the analysis for study objective 3.

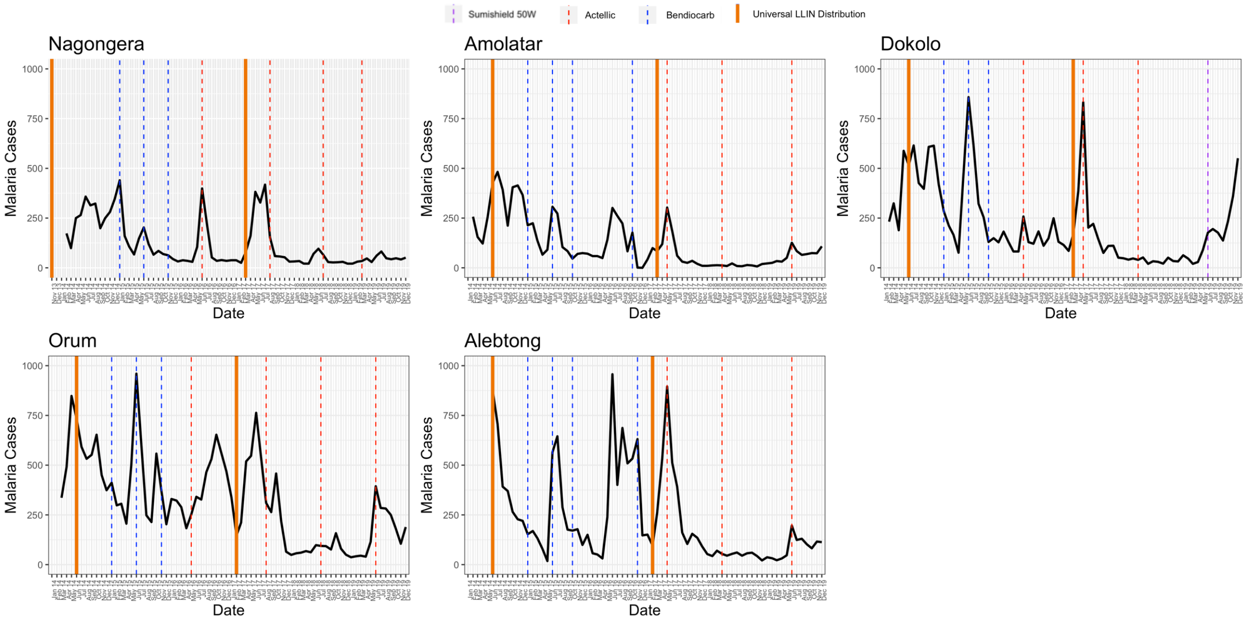

Supplementary Fig 7. Adjust IRR (the measure of center for the error bars) and 95% confidence intervals (the error bars) for study objective 3 with raw laboratory-confirmed case counts as model outcome.

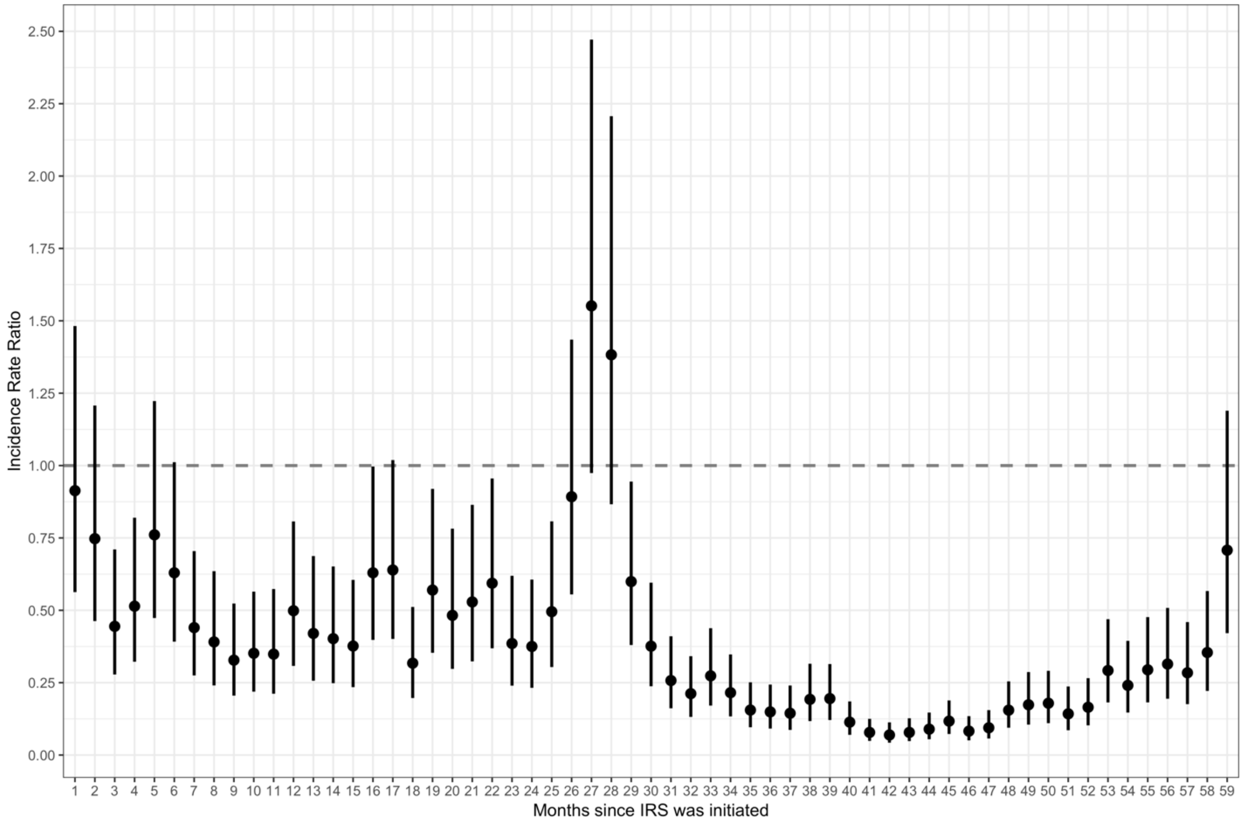

Supplementary Table 3. Coefficients and 95% confidence intervals for study objective 3. P-values are 2-sided and correspond to t-test from regression models. No adjustment for multiple comparisons was made.

Variable	**OR**	**pvalue**	**LL**	**UL**
Months since IRS initiated				
0	REF			
1	0.751	0.231	0.470	1.200
2	0.645	0.065	0.405	1.027
3	0.394	0.000	0.250	0.622
4	0.488	0.002	0.310	0.769
5	0.780	0.290	0.492	1.236
6	0.551	0.011	0.348	0.874
7	0.381	0.000	0.241	0.603
8	0.347	0.000	0.216	0.556
9	0.295	0.000	0.187	0.466
10	0.310	0.000	0.195	0.491
11	0.289	0.000	0.178	0.468
12	0.415	0.000	0.259	0.663
13	0.335	0.000	0.208	0.540
14	0.322	0.000	0.202	0.513
15	0.310	0.000	0.196	0.491
16	0.522	0.004	0.334	0.815
17	0.521	0.005	0.332	0.819
18	0.300	0.000	0.188	0.479
19	0.527	0.007	0.330	0.841
20	0.454	0.001	0.283	0.726
21	0.532	0.010	0.329	0.860
22	0.738	0.199	0.464	1.173
23	0.380	0.000	0.240	0.602
24	0.436	0.001	0.273	0.696
25	0.467	0.002	0.290	0.751
26	0.756	0.235	0.477	1.199
27	1.196	0.437	0.762	1.878
28	1.078	0.747	0.685	1.696
29	0.500	0.002	0.321	0.778
30	0.329	0.000	0.210	0.515
31	0.227	0.000	0.144	0.357
32	0.180	0.000	0.113	0.287
33	0.227	0.000	0.143	0.359
34	0.170	0.000	0.107	0.271
35	0.127	0.000	0.080	0.203
36	0.121	0.000	0.075	0.195
37	0.108	0.000	0.066	0.178
38	0.151	0.000	0.094	0.244
39	0.154	0.000	0.097	0.244
40	0.094	0.000	0.059	0.152
41	0.065	0.000	0.041	0.102
42	0.062	0.000	0.038	0.099
43	0.069	0.000	0.043	0.111

44	0.078	0.000	0.048	0.126
45	0.096	0.000	0.060	0.152
46	0.067	0.000	0.042	0.107
47	0.074	0.000	0.045	0.120
48	0.125	0.000	0.077	0.204
49	0.129	0.000	0.079	0.211
50	0.138	0.000	0.086	0.222
51	0.113	0.000	0.069	0.185
52	0.130	0.000	0.082	0.206
53	0.229	0.000	0.145	0.362
54	0.209	0.000	0.129	0.339
55	0.255	0.000	0.159	0.408
56	0.259	0.000	0.162	0.413
57	0.232	0.000	0.145	0.370
58	0.269	0.000	0.170	0.425
59	0.565	0.026	0.342	0.935
Precipitation (1 month lag)	1.000	0.995	0.990	1.010
Proportion of tests RDT	0.743	0.027	0.572	0.967
Count of patients not suspected of having malaria	1.000	0.018	1.000	1.000
Calendar month				
January	REF			
February	0.865	0.415	0.610	1.226
March	0.801	0.257	0.547	1.175
April	0.780	0.209	0.529	1.150
May	1.517	0.035	1.031	2.233
June	2.357	0.000	1.599	3.476
July	2.248	0.000	1.504	3.360
August	1.505	0.064	0.977	2.319
September	1.281	0.235	0.851	1.927
October	1.613	0.018	1.087	2.394
November	1.528	0.030	1.042	2.239
December	1.103	0.581	0.779	1.563
Constant	536.361	0.000	308.828	931.531

Supplementary Table 4. Details of indoor residual spraying campaigns for 5 districts that initiated IRS in 2013-14.

**MRC**	**District**		**1st round**	**2nd round**	**3rd round**	**4th round**	**5th round**	**6th round**	**7th round**
Nagongera HCIV	Tororo	*Insecticide*	Bendiocarb	Bendiocarb	Bendiocarb	Actellic	Actellic	Actellic	Actellic
		Date	08-Dec-14 to 19-Feb-15	08-Jun-15 to 12-Jul-15	02-Nov-15 to 12-Dec-15	12-Jun-16 to 9-Jul-16	17-July-17 to 19-Aug-17	11-Jun-18 to 27-7-18	18-Mar-19 to 15-Apr-19
		Coverage	N/A	94.6%	96.3%	93%	94.9%	95.8%	92.1%
Amolatar HCIV	Amolatar	*Insecticide*	Bendiocarb	Bendiocarb	Bendiocarb	Bendiocarb	Actellic	Actellic	Actellic
		Date	08-Dec-14 to 19-Feb-15	08-Jun-15 to 12-Jul-15	12-Oct-15 to 07-Nov-15	24-Oct-16 to 19-Nov-16	2-May-17 to 6-June-17	9-Apr-18 to 12-May-18	27-May-19 to 27-June-19
		Coverage	N/A	96.1%	91.4%	94.1%	95.8%	96.1%	94.2%
Dokolo HCIV	Dokolo	*Insecticide*	Bendiocarb	Bendiocarb	Bendiocarb	Actellic	Actellic	Actellic	Sumishield 50W
		Date	08-Dec-14 to 19-Feb-15	08-Jun-15 to 12-Jul-15	12-Oct-15 to 07-Nov-15	18-Apr-16 to 9-Jul-16	2-May-17 to 6-June-17	9-Apr-18 to 12-May-18	27-May-19 to 27-June-19
		Coverage	N/A	88.7%	92.1%	94.8%	97.6%	94.3%	95.2%
Orum HCIV	Otuke	*Insecticide*	Bendiocarb	Bendiocarb	Bendiocarb	Actellic	Actellic	Actellic	Actellic
		Date	08-Dec-14 to 19-Feb-15	08-Jun-15 to 12-Jul-15	12-Oct-15 to 07-Nov-15	18-Apr-16 to 9-Jul-16	17-July-17 to 19-Aug-17	11-Jun-18 to 27-7-18	27-May-19 to 27-June-19
		Coverage	N/A	96.6%	94.8%	97.6%	96.7%	98.4%	98.8%
Alebtong HCIV	Alebtong	*Insecticide*	Bendiocarb	Bendiocarb	Bendiocarb	Bendiocarb	Actellic	Actellic	Actellic
		Date	08-Dec-14 to 19-Feb-15	08-Jun-15 to 12-Jul-15	12-Oct-15 to 07-Nov-15	24-Oct-16 to 19-Nov-16	2-May-17 to 6-June-17	9-Apr-18 to 12-May-18	27-May-19 to 27-June-19
		Coverage	N/A	96.9%	95.4%	97.5%	98.3%	90.9%	95.0%

Coverage data not available for the first IRS campaign in 2014.
