# Supplementary material for: The impact of stopping and starting indoor residual spraying on malaria burden in Uganda
Source: Nat Commun. 2021 May 11;12:2635. doi: 10.1038/s41467-021-22896-5 (PMC8113470; doi:10.1038/s41467-021-22896-5)
Supplement: Supplementary file 3 — Reporting Summary [file 41467_2021_22896_MOESM3_ESM.pdf]

nature research

Corresponding author(s): Adrienne Epstein

Last updated by author(s): Mar 18, 2021

Reporting Summary

Nature Research wishes to improve the reproducibility of the work that we publish. This form provides structure for consistency and transparency in reporting. For further information on Nature Research policies, see our Editorial Policies and the Editorial Policy Checklist.

Statistics

For all statistical analyses, confirm that the following items are present in the figure legend, table legend, main text, or Methods section.

n/a	Confirmed	
☐	☒	The exact sample size (*n*) for each experimental group/condition, given as a discrete number and unit of measurement
☐	☒	A statement on whether measurements were taken from distinct samples or whether the same sample was measured repeatedly
☐	☒	The statistical test(s) used AND whether they are one- or two-sided<br>*Only common tests should be described solely by name; describe more complex techniques in the Methods section.*
☐	☒	A description of all covariates tested
☐	☒	A description of any assumptions or corrections, such as tests of normality and adjustment for multiple comparisons
☐	☒	A full description of the statistical parameters including central tendency (e.g. means) or other basic estimates (e.g. regression coefficient) AND variation (e.g. standard deviation) or associated estimates of uncertainty (e.g. confidence intervals)
☐	☒	For null hypothesis testing, the test statistic (e.g. *F*, *t*, *r*) with confidence intervals, effect sizes, degrees of freedom and *P* value noted<br>*Give P values as exact values whenever suitable.*
☒	☐	For Bayesian analysis, information on the choice of priors and Markov chain Monte Carlo settings
☐	☒	For hierarchical and complex designs, identification of the appropriate level for tests and full reporting of outcomes
☐	☒	Estimates of effect sizes (e.g. Cohen's *d*, Pearson's *r*), indicating how they were calculated

Our web collection on statistics for biologists contains articles on many of the points above.

Software and code

Policy information about availability of computer code

Data collection	Data were collected using MS Access v16.0 (Microsoft Corporation, Redmond, WA).
Data analysis	We used R v3.6 and Stata v14. Computing code is available at https://doi.org/10.5281/zenodo.4625804

For manuscripts utilizing custom algorithms or software that are central to the research but not yet described in published literature, software must be made available to editors and reviewers. We strongly encourage code deposition in a community repository (e.g. GitHub). See the Nature Research guidelines for submitting code & software for further information.

Data

Policy information about availability of data

All manuscripts must include a data availability statement. This statement should provide the following information, where applicable:
- Accession codes, unique identifiers, or web links for publicly available datasets
- A list of figures that have associated raw data
- A description of any restrictions on data availability

The datasets generated during and/or analysed during the current study are available in the github repository https://doi.org/10.5281/zenodo.4625804

Field-specific reporting

Behavioural & social sciences study design

All studies must disclose on these points even when the disclosure is negative.

Study description	Quantitative analysis of enhanced surveillance health facility data
Research sample	This analysis used outpatient data from 14 sentinel health facilities across Uganda. All outpatients seen during the study period were included in the analysis (n=1,657,826 outpatient visits of whom 416,928 tested positive for laboratory confirmed malaria). The mean age of patients was 22.5 (SD 19.0) and 33.2% of patients were male. This is a convenience sample and is therefore not representative. This sample is justified as it allows for measurement of changes in malaria incidence over time (the goal of this study), assuming stable population size and care seeking patterns.
Sampling strategy	The sample is a convenience sample of outpatients. No sample size calculations were used as this was a secondary data analysis of health facility data. All outpatients seen during the study window and testing positive for malaria were included in the analysis.
Data collection	At each sentinel health facility, individual-level outpatient department records are entered into an electronic MS Access v16.0 (Microsoft Corporation, Redmond, WA) database for all individuals presenting to the outpatient departments of the health facilities using a standardized format. Data collected includes patient demographics (age, gender, and village of residence), results of laboratory tests (rapid diagnostic test or microscopy), diagnoses given, and treatments prescribed. This data comes from the HMIS 031 standardized form. The Health Information Assistant at each public health facility uses HMIS outpatient registers to input the data into the Access database. These data entrants are not blinded to the treatment (IRS) status. Data are sent to the Uganda Malaria Surveillance Program data center and cleaned before transfer to Stata (Stata Corp, College Station, TX) v14 for analysis.
Timing	April 1, 2013-December 31, 2019
Data exclusions	No data were excluded from the analysis.
Non-participation	This is a secondary analysis of de-identified health facility surveillance data. No participants dropped out/declined participation.
Randomization	Participants were not randomized to treatment groups. Instead, we attempted to control for variables that may impact changes in malaria incidence, care seeking, and case reporting including: monthly rainfall at the health facility lagged by 1 month extracted from the Climate Hazards Infrared Precipitation with Stations database, indicator variables for month of the year (to adjust for seasonal effects), the proportion of tests that were RDTs in that month (vs. microscopy), and the number of individuals who attended the health facility but were not suspected of having malaria in that month (to adjust for potential changes in care-seeking behaviors over time).

Reporting for specific materials, systems and methods

We require information from authors about some types of materials, experimental systems and methods used in many studies. Here, indicate whether each material, system or method listed is relevant to your study. If you are not sure if a list item applies to your research, read the appropriate section before selecting a response.

Materials & experimental systems

n/a	Involved in the study
☒	☐ Antibodies
☒	☐ Eukaryotic cell lines
☒	☐ Palaeontology and archaeology
☒	☐ Animals and other organisms
☐	☒ Human research participants
☒	☐ Clinical data
☒	☐ Dual use research of concern

Methods

n/a	Involved in the study
☒	☐ ChIP-seq
☒	☐ Flow cytometry
☒	☐ MRI-based neuroimaging

Human research participants

Policy information about studies involving human research participants

Population characteristics	See above.
Recruitment	This analysis uses a convenience sample of outpatients that attended surveillance health facilities over the study window. There was no participant recruitment.
Ethics oversight	Ethical approval for study procedures and data collection was provided by ethics committees of University of California San Francisco (REF 250046), the School of Medicine College of Health Sciences at Makerere University (REF 2019-087), and Uganda National Council of Science and Technology (REF HS 2659). Written informed consent was not required by the ethical review committees due to the routine, de-identified nature of the data.

Note that full information on the approval of the study protocol must also be provided in the manuscript.
